# A planetary boundaries perspective on the sustainability: resilience relationship in the Kenyan tea supply chain

**DOI:** 10.1007/s10479-021-04096-y

**Published:** 2021-05-18

**Authors:** George Mutugu Mwangi, Stella Despoudi, Oscar Rodriguez Espindola, Konstantina Spanaki, Thanos Papadopoulos

**Affiliations:** 1 Lotus Consulting, Nairobi, Kenya; 2 Aston Business School, Aston University, Birmingham, UK; 3 Loughborough School of Business and Economics, Loughborough University, Loughborough, UK; 4 Kent Business School, University of Kent, Canterbury, UK

**Keywords:** Planetary boundaries, Resilience, Sustainability, Agriculture, Producers

## Abstract

The purpose of this paper is to examine whether agricultural supply chains (ASC) can be simultaneously sustainable and resilient to ecological disruptions, using the Planetary Boundaries theory. The nine different Planetary Boundaries i.e. climatic change, biodiversity loss, biogeochemical, ocean acidification, land use, freshwater availability, stratosphere ozone depletion, atmospheric aerosols and chemical pollution are examined in relation to ASC sustainability and resilience. Kenya’s tea upstream supply chain sustainability and resilience from the ecological point of view is questioned. This study adopts a multi-case study analysis approach of nine producer organisations from Kenya’s tea supply chain. The data from the in-depth semi-structured interviews and a focus group discussion are analysed using thematic analysis. The Kenyan tea supply chain producers are not aware of all the nine planetary boundaries, although these impact on their resilience practices. They are engaged in pursuing both sustainability and resilience practices. They implement mainly environmental practices in relation to sustainability, while only a few of them are implementing resilience practices. The sustainability and resilience concepts were found to be interrelated, but resilience does not improve at the same pace as sustainability. It is suggested that the relationship between sustainability and resilience is non-linear. Limitations and future research avenues are also provided.

## Introduction

The last few decades have seen increased interconnectedness among suppliers and manufacturers resulting in both high supply chain complexity and dependency among firms (Blackhurst et al., 2005; Christopher & Holweg, 2011). Even though this has resulted in increased efficiency, it has also left organizations exposed to supply chain interruptions because the current turbulence and uncertainty in global markets renders traditional risk approaches to risk management impotent (Pettit et al., 2013). The effect can be undesirable losses to the organization (Ponomarov & Holcomb, 2009). At the same time, stakeholders are increasingly demanding that organizations conduct their business operations socially and in an environmental and ethical manner (Carter & Easton, 2011). Integrating sustainability measures into business operations has gained strategic importance (Fahimnia & Jabbarzadeh, 2016). Hence, organizations need to adopt proactive measures to enhance the resilience of the supply chain whilst ensuring sustainability of their operations (Ponomarov & Holcomb, 2009).

While both sustainability and resilience have been discussed in the broader Supply Chain Management (SCM) tenets, their interrelationship in the Agricultural Supply Chain (ASC) has received little attention (Leat & Revoredo-Giha, 2013; Michel-Villarreal et al., 2019). This neglect is unfortunate because the expected world population growth combined with socio ecological factors adds even more pressure to ASCs (Despoudi, 2021; Spanaki et al., 2021; Stone et al., 2015a). Even though organizations have embarked on implementing various sustainability practices there is limited knowledge about the broader effects of sustainability practices employed in organizations on the supply chain ability to endure disruptions (Fahimnia & Jabbarzadeh, 2016). Pre-existing research on the ASC focuses either on sustainability or resilience practices (Bajželj et al., 2020; Colwill et al., 2016; Despoudi, 2020; Manning & Soon, 2016; Soussana, 2014; Stone & Rahimifard, 2018). However, there is no clarity about their possible interrelationship in the ASC (Ambler-Edwards et al., 2009; Fahimnia & Jabbarzadeh, 2016).

Current conceptualizations of the relationship between resilience and sustainability are inconclusive as they focus on either the focal firm or the overreaching industry and the broader global context tends to be ignored (Whiteman et al., 2012). Micro-level resilience involves the direct engagement between buyers and suppliers to mitigate disruptions, whereas macro-level resilience focuses on the collaboration of different stakeholders in the industry including corporations, governments or trade associations (Azadegan & Dooley, 2021). Both levels can deliver valuable insights. However, the current climatic crisis requires a broader systems perspective, which integrates micro, meso and macro level factors such as the planetary boundaries (Huang & Farboudi Jahromi, 2021; Prentice et al., 2021). Prentice et al. (2021) and Azadegan and Dooley (2021) argue that it is important to consider the meso level, which is about promoting collaboration between firms from different supply networks. Micro-level resilience struggles in instances when major disruptions affect both supply and demand, whereas the macro-level is more focused on medium to long term response and can have a stable nature that can complicate collaboration (Azadegan & Dooley, 2021). Hence, the meso level can be useful to connect the micro and macro levels and enable a better response to sustainability and resilience challenges (Sievers-Glotzbach & Tschersich, 2019). Although several research has focused on the micro-level, more analysis is needed at the meso and macro levels connecting the three as all three of them operate under the same system and limits i.e. planetary boundaries (Shaw et al., 2018). Indeed, the link between sustainability and resilience needs to consider local interactions, responsive structures inclusive of different supply networks, global collaboration with industry and governmental stakeholders, and the overall planetary system and its respective limits (Azadegan & Dooley, 2021; Goworek et al., 2018; Matthews et al., 2014)

Kenya’s Tea Supply Chain (KTSC) is facing vulnerabilities from climatic change that threaten tea productivity, while at the same time reduce the amount of land available for tea production (Hadyniak, 2014). Kenya is based in a region that is expected to be significantly affected by climatic change according to the United Nation’s (UN) assessment on climate change (Hadyniak, 2014). Previous studies addressing climatic change and current interventions in the tea industry in Kenya (Cracknell, 2015; Kabubo-Mariara & Karanja, 2007; Ochieng et al., 2016) made recommendations for adaptation without reflecting on the way these adaptability recommendations affect both the resilience and sustainability of the industry. For instance, Cracknell (2015), proposes that the industry could adapt better by replacing the current bushes with drought and frost resistant clones. However, such an approach would not necessarily increase resilience, as other factors could impact growth and thereby compromise the financial sustainability of the industry as tea takes about 5–7 years to mature (Chang & Brattlof, 2015). Thus, the study of sustainability and resilience of KTSC from an ecological point of view is warranted. This research explores the sustainability—resilience relationship in the ASC context in the face of projected disruptions using the lens of the planetary boundaries theory (PBT), which draws upon the natural sciences to incorporate micro, meso and macro level factors (Rockström et al., 2009; Whiteman et al., 2012). The study is using the KTSC as case study to address the problematic experienced in the country and provide insights informing theory and practice. Specifically, this paper aims to examine whether the KTSC can simultaneously be sustainable and resilient under the PBP using semi-structured interviews and a focus group with practitioners. Thematic analysis was undertaken to identify the main themes and develop theoretical and managerial implications. To fulfil the overall aim of this research, the research questions are as follows:

- RQ1: what are the current levels of planetary boundaries as well as sustainability and resilience levels in the Kenyan Tea Supply Chain from the producers’ perspective?
- RQ1: what is the relationship between sustainability and resilience under the planetary boundaries’ theory in the Kenyan Tea Supply Chain from the producers’ perspective?

As a result of human activities, we have already surpassed the climatic change, biodiversity loss and biogeochemical safe zones (Matthews et al., 2016). This is particularly problematic for agricultural supply chains (ASCs), because these are based on resource-intensive activities. Considering that PBT acknowledges the interconnectedness of different issues and the impossibility to address one of them without influencing the others (Whiteman et al., 2012), it is essential to identify the planetary boundaries encountered in ASCs to develop holistic policies able to make impactful improvements considering the relationship between them. Hence, the first step of this research involves ascertaining the current awareness levels of the KTSC producers about the nine planetary boundaries, which is encapsulated on RQ1.

Having more understanding about those boundaries, it is possible to delve into the relationship between sustainability and resilience. The lack of consensus on the literature about their relationship (Marchese et al., 2018) endangers implementing policy and guidelines that could inadvertently have negative effects in either one of those dimensions or risking having less impactful results. Therefore, their link needs to be addressed through deeper investigation examining their interaction to inform research and practice. This research tackles that need as part of RQ2. The relationship of sustainability and resilience practices is examined through an analysis of current practices and the effect of them on both dimensions to provide more clarity in the context of the KTSC from the producers’ perspective. Having determined the latter relationship, relationship between sustainability and resilience will be explored in relation to the factors that will emerge from the PBT.

Therefore, the contribution of this research to the current body of knowledge is threefold. It analyses practices promoting sustainability and resilience from Kenya’s tea upstream supply chain using the lens of PBT, an approach never undertaken before. It investigates the link between sustainability and resilience which is currently underexplored and without a clear consensus (Ambler-Edwards et al., 2009; Fahimnia & Jabbarzadeh, 2016). Finally, this research accounts for micro, meso and macro factors affecting sustainability and resilience to disruptions in the ASC. These contributions can be used to inform tea supply chains in similar countries and to inform similar studies in different countries to develop analytical generalisations (Tsang, 2014).

This paper is organized in six sections. Section 1 presents the literature review and the theoretical background of this research. Section 3 outlines the research methodology employed in this study, while Sect. 4 presents the findings of this research. Finally, Sect. 5 is the discussion and Sect. 6 the conclusions with the research implications and future research directions.

## Literature review and theoretical background

This section reviews research on resilience and sustainability in the supply chain and in ASC. It provides an overview of the different definitions and practices in supply chain sustainability and sustainability in AGS, the concept of supply chain resilience and ASC resilience, and the relationship between sustainability, resilience and ASC is presented. Additionally, the theorical lens of this study which is the PBP theory is explained along with an overview of Kenya’s tea supply chain.

### Supply chain sustainability and agricultural supply chain sustainability

Even though the term sustainability has gained increased prominence, there is neither consensus on its definition nor how to operationalize (De Silva & Forbes, 2016). However, the World Commission on Environmental and Development defines sustainability as “meeting the needs of the present without compromising the ability of future generations to meet their own needs” (Commission on Environment, 1987, p. 8). Sustainability has been described as a moral value question that should address the preservation of the abilities of future generations (Matthews et al., 2016), but current business practices have failed to live up to this billing as they still focus on win–win outcomes that tend to favour the financial objective of the firms (Golicic & Smith, 2013). Dubey and Gunasekaran (2016) argue that sustainable supply chain design needs to be agile, adaptable and aligned.

Sustainability has been considered as one of the main capabilities of organisations, which further creates a competitive advantage to the firm (Shibin et al., 2020). To benefit from the advantages of sustainability, organizations have to acknowledge their social and environmental impacts at each stage of production (Koplin, 2005) as companies have recently come under pressure from both regulators and consumers for a lack of sustainability compliance (Seuring & Müller, 2008). In the ASC context sustainability is defined as food is produced, processed and traded in ways that: contributes positively to local economies, protects diversity of plants and animals and natural resources, helps to manage climatic change, and provides social benefits to consumers (Sustain, 2015).

SustainAbility (2011) defines ASC sustainability as having a reliable, resilient and transparent supply chain, which produces food within ecological limits, empowers food producers, and ensures accessible and nutritious food for all. In the HM UK government report the ‘Food 2030’ (2010), it is stated that sustainable food is food that is produced, processed and distributed to feed a growing global population in ways which use global natural resources sustainably, enable the continuing provision of the benefits and services, ensure a healthy natural environment provides, promote high standards of animal and welfare, protect food safety, and make significant contribution to rural communities. Therefore, current projections of population growth to 9 billion people by the year 2050 (Kastner et al., 2012) combined with climate change and the increasing number of disruptions on supply chains, make it essential to ensure sustainable supply chains are implemented to deliver food to regions under different conditions, especially in the face of disruptions (Schiffling et al., 2020; Soussana, 2014). Despite the increased attention and academic research focused on sustainability (Seuring & Müller, 2008), there still critical issues that need to be addressed to enable business leaders achieve sustainability both at the strategic and tactical level (Pagell & Wu, 2009).

Existing academic work has focused on investigating social, economic and environmental factors individually ignoring an integrated approach (Gold et al., 2010). For instance, the study by Hutchins and Sutherland (2008) examined both social and environmental factors while Dewangga et al. (2008) examined social and environmental factors respectively. Additionally, most researchers have focused on developing sustainability measurement models that assess the sustainability of a system (Collins et al., 2010). For instance, Pullman et al. (2010) note that although researchers are free to test different practices, some practices are only industry specific and different practices have been identified by different researchers. The current research embraces the concept of all-round approach of sustainability (Despoudi, 2020; Elkington, 1998), which suggests ASC sustainability is about having the resources and the capabilities in the supply chain to create sustainable food consistently for now and for the future by balancing all three sustainability elements (i.e. environmental, social, and economic).

Environmental sustainability seeks to encourage practices that preserve the environmental resources of future generations addressing the efficient use of energy, reducing greenhouse gasses emissions, water management, waste management and lessen any ecological effects (Alhaddi, 2015; Goel, 2010). According to Despoudi (2020), environmentally sustainable ASC companies promote the responsible consumption of resources, managing their carbon footprints and openly informing the public about their performance. As regulations tighten, a common trend has seen less sustainable companies relocate or outsource their production function to other countries that have relaxed environmental restrictions (Alhaddi, 2015). For example, the past decade has seen a lot of ASC businesses moving their manufacturing to the far east and environmental compliance has been one of the reasons why they moved.

Social sustainability seeks to address fair labour and human practices that provide value and are beneficial to the community and may include, safeguarding against child labour, discrimination, fair compensation and promoting health and safety practices for its workers (Elkington, 1998). Large corporations in the ASC have been blamed for their social sustainability practices as they take advantage of small-scale producers by giving them unfair prices (Despoudi et al., 2020). ASC companies should benefit the community in which they operate as they consume their resources, and this may affect the livelihoods of the local population. Economic sustainability is related to the capabilities of the organisation to survive and progress in a way that will still supports the livelihoods of future generation, therefore giving emphasis to the long term financial strength (Alhaddi, 2015). Different indicators are taken into account which may include revenue growth, share price, supply chain costs and profitability while at the same time keeping social and environmental factors in control (Delai & Takahashi, 2011). The ASC from the producers’ perspective is facing economic sustainability issues (Papaioannou et al., 2020).

### Supply chain resilience and agricultural supply chain resilience

The concept of resilience was first introduced by Christopher and Peck (2004), when the world was recovering from the foot-and-mouth outbreak in the UK and the 9/11 bomb in America, both of which caused huge disruptions to global supply chains. Since then, there has been a steady growth in the amount of research in the field (Jüttner & Maklan, 2011; Kim et al., 2015). Nevertheless, Ponomarov and Holcomb (2009) noted that the idea is still in its exploratory stage. The concept of resilience has evolved through time (Behl & Dutta, 2019a, b). Christopher and Peck (2004) describe resilience as the “capacity of a supply chain to cope with the consequences of vulnerabilities and to get back to its original state or an even more desirable state once it is disrupted” Christopher and Peck (2004, p. 2). Later on, Ponomarov and Holcomb (2009) on the other hand, described it as an adaptive capability to prepare for unexpected eventualities respond and recover while at the same time maintaining a continuation of operations at a desirable level. Implicit in its definition is the ability to be adaptable and to absorb shocks that may engulf it (Folke, 2006).

While most definitions on resilience tend to favour a return to the original operating capacity or state of equilibrium, in a field as complex as food systems where social, economic, environmental and political issues define the space, it’s almost impossible to have this equilibrium (Stone & Rahimifard, 2018). Thus, considering that supply chain must incorporate the resilience concept to deal with the challenges posed by any disruptions (Queiroz et al., 2020), research on ASC resilience has been seen to support an adaptable system that is able to maintain “function” and adapt in the face of disruptions focus on maintaining an unbroken flow of safe and fitting food to consumers (Tendall et al., 2015). ASC resilience is of crucial importance because food, nutritional outcomes, livelihoods, and many other essential life-supporting services (Barrett et al., 2001). This was evident during the first wave of the COVID-19 pandemic in which ASCs failed to adapt and there were food shortages with many people not having access to food (Galanakis, 2020). According to Vroegindewey and Hodbod (2018) ASC resilience is about having the capacity to continue operations and services in the face of supply and demand disturbances through preparation, response, recovery and adaptation to changes.

A supply chain that possesses resilience capabilities is better able to withstand the unpredictability that modern supply chains must contend with because of the embedded adaptability and responsiveness (Kwak et al., 2018). At the same time, it can help achieve a competitive advantage by being able to recover faster than competitors from disruptions (Leat & Revoredo-Giha, 2013). The potential of resilience to support competitive advantage in organisations is essential, as industries are becoming more global and SMEs are becoming an important part of international value chains (Gunasekaran et al., 2011). Using the resource-based theory lens, Kwak et al. (2018) employ factor analysis and structural equation modelling in a sample of 174 South Korean manufacturers to explore the link between supply chain innovation, robustness an resilience, and competitive advantage. Their findings highlight the role of innovation to develop resilience and robustness, which in turn have a significant effect on the level of competitive advantage of the company.

Results from Abeysekara et al. (2019) agree with the importance of resilience to achieve competitive advantage. The study undertakes a dynamic capability perspective to investigate the way organisational assets and resources can support achieving high levels of performance and competitive advantage. The article introduces risk management, re-engineering, agility, and collaboration as dimensions of supply chain resilience. Partial least squares structural equation modelling is used to analyse 89 responses from Sri Lankan apparel manufacturers. Their results show the influence of agility and collaboration on competitive advantage. Hence, organisations need to consider the potential of resilience to support competitive advantage in the organisation. The importance of resilience is also highlighted in the study of Altay et al. (2018) who found that resilience can affect supply chain performance.

**Fig. 1:**
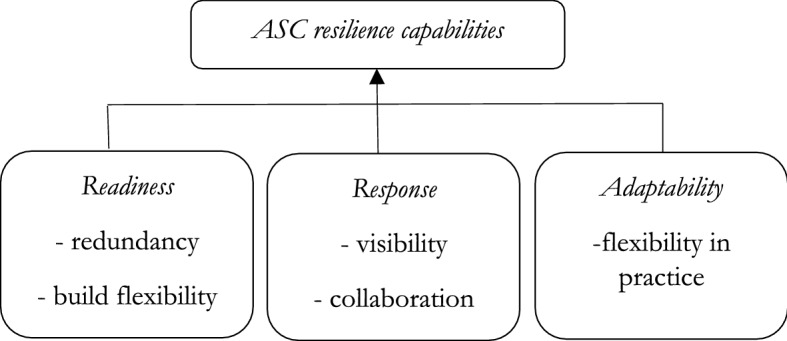
Capabilities for ASC resilience. *Source*: authors

The current business practices employed by supply chain managers in the pursuit of lower costs and creation of value for their shareholders greatly undermine the resilience capabilities of their supply chains. For instance, a lack of safety stock held in the ASC as well as a lack of back up capacity greatly reduces the abilities of a firm to retain its operational structure in the face of disruption (Pettit et al., 2010). Therefore, to be able to continuously deliver safe and fit food for consumption despite disruptions, ASCs should be able to adapt to prevailing situations by adopting suitable capabilities (Smith et al., 2016). This study adopts the three main themes of disruption as identified by Ponomarov and Holcomb (2009), and an additional stage suggested by Stone and Rahimifard (2018) to form the basis for grouping the core capabilities needed by a resilient ASC system. These are readiness, responsiveness and recovery, and adaptive strategy. This phases have also been referred to as resist, recover and adapt (Fahimnia & Jabbarzadeh, 2016; Leat & Revoredo-Giha, 2013). Figure 1 presents the different capabilities needed for ASC resilience.

Also described as the resistance stage (Fahimnia & Jabbarzadeh, 2016; Leat & Revoredo-Giha, 2013), it focuses on how an organization anticipates either by preparing for the disruption or avoiding the disruption. These elements help to identify and monitor changes in the environment as well as those elements that need to be built early to be utilised in other stages (Stone & Rahimifard, 2018). The readiness phase is about redundancy. Redundancy is the strategic use of built up extra capacity and stocks of agricultural products that can be used in a moment of emergency to provide cover before the systems restores for example, exponential demand could be catered for using spare stocks (Christopher & Peck, 2004). Holding spare capacity has however been criticized as an expensive way of doing business especially as business leaders try to reduce non-value adding activities in the supply chain (Pettit et al., 2013). In ASCs the food shelf life of particular products at times works against this particular capabilities usefulness (Stone et al., 2015b). The readiness phase also involves building flexibility as a core component. Flexibility is the ability of a supply chain to respond to changing business environment and customer requests (Lam & Bai, 2016). Flexibility can be used to enhance resilience in the ASC in several ways. For example; postponement, flexible transport systems, flexible labour arrangements, flexible order fulfilment (Christopher & Holweg, 2011; Pettit et al., 2013). Although flexibility helps ASCs to respond rapidly and recover, this can be enhanced by the existence of spare capacity or alternatives (redundancy) such as spare suppliers (Sheffi & Rice, 2005).

The response stage is also described as the recovery stage (Fahimnia & Jabbarzadeh, 2016; Leat & Revoredo-Giha, 2013). Response concerns pre-planned elements that mitigate impact of a disruption and at the same time help the system to remain functional (Stone & Rahimifard, 2018). The first response capability is visibility. Visibility concerns being able to see through structures and processes from one end of the supply chain to the other (Dubey et al., 2020). This will usually involve information being shared across the ASC by partners to enable the right knowledge to be utilised (Jüttner et al., 2003). Therefore, with proper information and knowing what is happening in the environment, organizations are thus able to respond better.

The second response capability is collaboration, which represents the glue that holds organizational relationships together when facing disruptions (Faisal et al., 2006). Collaboration is the building of partnerships to achieve mutual goals and further reducing uncertainty by distributing risk among partners (Despoudi et al., 2018; Pettit et al., 2010). In the ASC key collaborations need to be built among producers and their buyers to ensure food products sustainability (Despoudi et al., 2020). The adaptability stage involves the way in which the system is able to grow out of the disruption (Hohenstein et al., 2015). Adaptability could be defined as the capability of a system to adjust operations in response to certain eventualities by using different technology, reducing the lead time and learning from the experience (Dubey et al., 2018; Rasouli, 2019). In ASCs, this can be achieved by building flexibility into both the sourcing or the order fulfilment (Stone et al., 2015b). In sourcing, this could be achieved by having numerous sourcing options such as suppliers from different countries. Although this could limit the kind of relationships the organization is able to build, flexibility in order fulfilment could be achieved by having different distribution channels available to the organization (Manning & Soon, 2016).

### The sustainability and resilience relationship

Food security is essential for the welfare of populations and countries (Soussana, 2014). ASCs compared with other supply chains have traditionally experienced a higher level of uncertainty stemming from the short shelf life of food, long production throughput times, variability in quality and susceptibility to deterioration as raw materials move down the supply chain (Christopher & Holweg, 2011; Stone et al., 2015a). This situation is currently being aggravated by the unprecedented amount of dependency among partners further increasing the vulnerability on supply chains (Sheffi & Rice, 2005). ASCs are under increasing stress due to the increased amount of extreme weather conditions and geological activity attributed to climate change (Hodges et al., 2010). This climate related events are expected to reduce the amount of favourable land to farm and have a negative effect on food production and distribution (Suweis et al., 2015).

Findings from the Agriculture, Food Security and Climate Change forum outline the research priorities for sustainable ASCs (Vermeulen et al., 2010). These include the importance of strengthening European agriculture to reduce the increasing pressure of food production in other regions, reduce the environmental impact of agriculture, and build resilience to climate change in agricultural supply chains (Soussana, 2014). Sustainability has been a very popular topic, but resilience is emerging as a new crucial area in agri-food which is related to sustainability (Barbosa, 2021). In fact, a stocktaking exercise in 24 countries from 1990 to 2015 with participation of FAO, EU, WFP and the International Fund for Agricultural dimension found that agriculture must support environmental sustainability to make more resilient systems (Kennedy et al., 2020). Hence, achieving those aims require ASCs to consider the integration of sustainability, and resilience, especially in urgent and chaotic settings caused by disruptions (Queiroz et al., 2020; Venkatesh et al., 2019). That is the reason recent studies have shown the intersection between sustainability and resilience (Dolgui et al., 2020).

Understanding and finding ways to evaluate sustainability and resilience are key in ASCs for the society as a whole (Michel-Villarreal et al., 2019), because rather than just adapting, food supply chains have to create the conditions to deliver products under various circumstances (Schiffling et al., 2020). It allows to balance human requirements with necessary ecosystem functions for the survival of agricultural resources (Vroegindewey & Hodbod, 2018). Derissen et al. (2011) note that several studies on resilience and sustainability in supply chain management have considered each concept separately. Yet the influence between the two concepts on each other is too much to warrant ignoring one or the other. Fahimnia and Jabbarzadeh (2016) state that it is unrealistic to investigate one and fail to query how they affect each other. Both resilience and sustainability represent complex yet very important universal issues.

Although in social sciences the concepts of sustainability and resilience have been used interchangeably (Lamine, 2015), each one of them is valuable on its own right and they are essential elements in robust agricultural supply chains. Considering social, financial and environmental aspects, sustainability creates the capacity to survive. Companies nowadays, however, need to couple that concept with the capability to reinvent themselves when facing extreme challenges to thrive and move forward (Edgeman & Wu, 2016), which is the reason why the concept of resilience appeared more recently (Lamine, 2015). The implementation of both concepts, however, needs to be cautiously assessed because an inadequate understanding of their similarities and variances can result to underperformance (Marchese et al., 2018).

There are different views about how the sustainability and resilience concepts interact. An assessment on the literature by Marchese et al. (2018) came up with three broad perspectives on how these concepts interact with each other. However, this study suggests that there are four different perspectives about the sustainability—resilience relationship (Table 1). The first perspective suggests that resilience is an element of sustainability (Stone & Rahimifard, 2018), meaning that “increasing the resilience of a system makes that system more sustainable, but increasing the sustainability of a system does not necessarily make it more resilient” (Marchese et al., 2018, p. 1276). Sustainability in this case provides the systems objectives while resilience capabilities are merely there to achieve these objectives (Anderies et al., 2013). Papadopoulos et al. (2017) undertake an analysis of unstructured big data gathered online and structured data from 205 responses from managers involved in the Nepal earthquake in 2015 to investigate the role of big data for sustainability. They argue that using unstructured big data analysis can be used to develop frameworks for supply chain resilience and promote sustainability.

**Table 1:** The different perspectives about the relationship between sustainability and resilience. *Source*: authors

Perspective 1	Perspective 2
Resilience as element of sustainability	Sustainability as element of resilience
Increased sustainability no impact on resilience	Increased resilience, no impact on sustainability

Perspective 3	Perspective 4
Sustainability and resilience are not related	Sustainability and resilience are interrelated concepts

In their systematic literature review about food system outcomes, Stefanovic et al. (2020) highlight that the aim food supply resilience is to ensure food security even in situations following disturbances and shocks, which is the reason why resilience is a desired aspect of sustainable food supply chains. Michel-Villarreal et al. (2019) mentions that resilience can be seen as an enabler of sustainability because it allows systems to adapt to new ways of managing agricultural and natural systems (King, 2008). Lengnick (2015) presents an analysis looking at vulnerability and resilience of food systems to investigate the sustainability of the U.S. food supply chain. Their analysis of the literature show that food systems relying on external or distant resources and specialized marketing, production and supply chains are more vulnerable to climate change, which affects their sustainability. Therefore, all of them conclude that increasing the resilience of the system is crucial to optimize sustainability (Higgins et al., 2010).

The second perspective addresses sustainability as an element of resilience which means that “increasing the sustainability of a system makes that system more resilient, but increasing the resilience of a system does not necessarily make that system more sustainable” (Marchese et al., 2018, p. 1276). The purpose of resilience in this case is to uphold a certain objective such as profit during and after a disruption, and with increased sustainability the functionality of the system becomes more resilient to interruptions (Bansal & DesJardine, 2014). For instance, by adopting sustainable practices (social, economic and environmental) an organization would be able to remain profitable during a period of disruptions. Dubey et al. (2019a, b) provide an analysis of antecedents of resilient supply chains with information from 250 manufacturing companies through the lens of the resource-based view and the relational view. Their framework identifies cooperation and trust as promoters of sustainable commitment and performance, which can promote resilience in the supply chain. Their result confirm that cooperation, trust and also visibility have a significant influence on supply chain resilience. Chowdhury et al. (2021) undertook a systematic literature review to explore the impact of that major disruption on the supply chain. The analysis of 74 papers unveiled that sustainability and resilience took a prominent role in supply chain studies accounting for the effects of the pandemic. The authors highlight the gap of looking at the impact of sustainable practices on resilience.

There is also evidence that investment in either sustainability or resilience is not necessarily associated with an increase on the other. The third research perspective views resilience and sustainability as different elements each with separate objectives (Michel-Villarreal et al., 2019). That means neither of the two components contribute towards the advancement of the other (Marchese et al., 2018) with policies potentially affecting one, both or none of them (Derissen et al., 2011; Lew et al., 2016). King (2008) contrast engineering resilience, ecological resilience and adaptive capacity resilience with alternative agri-ecological systems. The findings suggest that resilience can be essential to design these alternative systems.

The fourth perspective argues that the sustainability—resilience concepts are interrelated and in need of one another. Higgins et al. (2010) argue that both concepts are related and investigate the potential of operational research methodologies to enhance them. The authors focus on the value of agent-based modelling, dynamical systems modelling and network analysis to develop insights into ASCs. Hoy (2015) categorized sustainability as part of agroecosystem health and describes the Agroecosystems Management Program at the Ohio State University which uses an approach of promoting self-organizing social network behaviour and entrepreneurship through the development of online tools to generate diversity. Their experience shows the potential of managing diversity in agroecosystems and encouraging innovation as valuable steps towards agroecosystem health and resilience. Vieira et al. (2018) mention that urban food systems need to be sustainable in their practices and resilient to manage disruptions. They present a systematic literature review combining both concepts and highlight the importance of healthy food, connectivity between urban and rural areas, existence of a strong local food economy and food production, reduced food waste, and active participation of all actors in decision-making regarding UFS as relevant principles for sustainable and resilient systems.

Das (2019) argue that sustainable food supply chains need to include resilience criteria to cope with disturbances. They propose a deterministic optimization model aiming to maximize realization of food production and maximize profit. They highlight the importance of supply chain approaches such as green and lean to increase resilience and social responsibility. Zhu and Krikke (2020) study the impact of disruptions, specifically the COVID-19 contingency, on supply chain demand. The authors introduce a systems dynamics simulation model to show that the development of sustainable and resilient ASCs restricting the information sharing that causes endogenous demand and utilize a loosely coupled strategy for decision-making to reduce the negative influence of endogenous demand. Mohammed et al. (2021) coins the term “gresilient” to combine green and resilient practices. They integrate both with traditional practices for supplier selection. The weights of the criteria are obtained through AHP and the suppliers are evaluated using TOPSIS. Once both tools are employed, the results are used in a multi—objective resource allocation model. Ivanov (2020) argues that supply chains should be viable across three perspectives: agility, sustainability and resilience. They mention that viability involves the interaction of sustainability and resilience for coping with negative disturbances and recovering from short-term and long-term shocks involving societal and economic transformations. Cristiano (2021) uses the case of northern Italy to discuss the way that a horticultural collaborative production system can serve as basis to improve socio-economic sustainability and resilience in a region.

Although there is evidence that sustainability practices can promote resilience, Sarkis (2020) mentions that there are sustainability concerns about measures to build resilience. For instance, there has been significant discussion about the value of local supply to increase sustainability in areas affected by disruptions (Matopoulos et al., 2014). Schmitt et al. (2017) use information from 14 local and global suppliers to compare the results between groups looking at environmental, economic, social, health and ethical indicators. The outranking analysis employed shows that although local suppliers perform better in general, the result depends on factors beyond the distance of suppliers. Local and intermediate suppliers are strong in health and socio-economic aspects such as resilience, whereas global food suppliers outperform local and intermediate suppliers in affordability and climate change mitigation. On the other hand, sustainability has been associated with alignment of objectives and information sharing (Despoudi et al., 2018). However, Vilalta-Perdomo and Hingley (2018) provide an alternative view looking at food-micro producers more as a community rather than links in a chain using information from six in-depth interviews, an online survey, and telephone surveys. They highlight that collaboration does not necessarily require shared aims and support the use of collective action for individual improvements. Their results show that collective action can be important to build resilience in food-micro producers.

Additionally, Vroegindewey and Hodbod (2018) look at resilience from the perspective of socio-ecological systems. They highlight the importance of redundancies, managing connectivity between components, manage variables and feedback, flexibility, and connectivity as principles required for resilience building. Stone and Rahimifard (2018) agree with that view and outline flexibility, risk awareness, redundancy, early warning systems and security as core intra-organizational factors for resilience, whereas collaboration, flexibility, agility, visibility, adaptability, and redundancy were recognized as intra-supply chain factors. Nevertheless, redundancy can create a conflict between sustainability and resilience (Bajželj et al., 2020; Sarkis, 2020). Bajželj et al. (2020) focused on that trade-off by analyzing the effect of waste reduction strategies on different aspects of resilience. They analyzed literature to mapped reinforcing or opposing relationships between promoting sustainability and reducing food waste. Their findings suggest that redundancy can potentially enhance short-term resilience (e.g. over-production and over-purchasing), but with the risk of having no real impact in resilience if there are no ways to mitigate spoilage and have significant negative impact in sustainability of the system.

Sarkis (2020) in his analysis of supply chain management after COVID-19 mentions that building redundancy capacity and capabilities can lead to wasted resources and energy. Pavlov et al. (2019) looking at seaport operations identify that suboptimal network redundancy can affect the environment negatively, whereas insufficient redundancies can lead to high vulnerability. Hence, potential conflict of efficiency and resilience can be affecting the link between sustainability and resilience (Dolgui et al., 2020). Although the relationship between sustainability and resilience is yet to ascertained, Ambler-Edwards et al. (2009) observed that despite possible conflicts, future food systems will have to combine the two. Additionally, a study by Colwill et al. (2016) recommends that the long term ASC sustainability will only be achieved by coupling it with resilience. Indeed, the relationship between both concepts can be crucial (Jabbarzadeh et al., 2018), especially if there is a trade-off between them that needs to be addressed (Pavlov et al., 2019). At the strategic level, the contribution of Fahimnia et al. (2018) provide insights about the relationship between greenness and robustness. Their use of an environmental performance scoring approach and a robustness measure showed the way there can be instances in which sustainability and robustness can have a positive relationship or be in conflict. This trade-off has been addressed in the supply network design area by Jabbarzadeh et al. (2018). They introduce a stochastic bi-objective optimisation model to support outsourcing decisions and the selection of resilient strategies. The model aims to minimise cost and maximise sustainability in disruptions. The system is solved using a combination of MATLAB and GAMS for a case study of the plastic pipe industry.

But although there exists a lot of interest in the two concepts, what this means in the upstream stage of the ASC is still not understood (Leat & Revoredo-Giha, 2013).

### The Kenyan tea supply chain

The aforementioned issues are expected to disproportionately affect global economies, especially developing economies in African countries that are heavily dependent on agriculture. The significance of such trends will be high in Kenya where agriculture contributes an estimated 26% of the gross domestic product (GDP) and more than 80% employment of the total population (KNBS, 2018). The country’s agricultural sector is highly dependent on rain for cultivation of major food crops such as maize as well as foreign exchange earners like tea and coffee (Ochieng et al. 2016). Hence, climatic variability and change resulting in reduced and irregular rainfall leave the country exposed to loss of agricultural production and increased food insecurity (Ochieng et al., 2016). For instance, unfavourable weather conditions in the year 2017 substantially stifled production of key crops and caused drought that triggered a national emergency (Schmidt et al., 2017). Additionally, impacting the GDP growth rate from 5.9% in the year 2016 to 4.9% in 2017 (KNBS, 2018).

Kenya is the third largest producer of tea in the world and the biggest seller of black tea (Tea & Centre, 2012) with an export value estimated at USD 1.33 billion in the year 2013 (Chang & Brattlof, 2015). The sector is dominated by small scale producers (own about 4–8 ha of land, not entirely dedicated to tea production) accounting for more than 60% of the production as compared to estates that account for less than 40% of produce (Angelucci et al. 2013). Small scale producers through the support of the government own their own production facilities that process and market tea giving them a considerable advantage as compared to other tea producing countries (Angelucci et al., 2013). Nonetheless, despite the importance of the commodity to the country several challenges face the sector ranging from poor infrastructure, high production costs, declining global tea prices, insufficient research, low levels of value addition and climatic change (Hadyniak, 2014). Hence, the sustainability and resilience of KTSC requires further study to support regional development.

### Theoretical lens: the planetary boundaries perspective (PBP)

“The planet’s environment has been unusually stable for the past 10,000 years…” (Rockström et al., 2009, p. 472). However, trends are changing and there is evidence that the system is losing its stability “now, largely because of a rapidly growing reliance on fossil fuels and industrialized forms of agriculture, human activities have reached a level that could damage the systems that keep earth in the desirable Holocene state” (Rockström et al., 2009, p. 472). Despite awareness of the deteriorating state of the ecosystem, researchers have yet to link business operations to the global ecological processes (Whiteman et al., 2012), with existing theories warranting a paradigm shift to address the already dilapidated ecological sphere (Matthews et al., 2016).

The planetary boundaries approach is a social-ecological system that recognizes that one issue alone (e.g., climate change) cannot be managed individually without influencing the other planetary perspectives as they are determined by a set of inter connected social and environmental processes (Whiteman et al., 2012). Rockström et al. (2009) introduced a set of nine boundaries to quantify the safe limits that the earth can safely live in and outside of which the earth cannot function normally. These boundaries include; climate change, biodiversity loss, biogeochemical, ocean acidification, land use, freshwater availability, stratosphere ozone depletion, atmospheric aerosols and chemical pollution.

We have already crossed beyond the safe zone for three boundaries, namely climate change, biodiversity loss and biogeochemical; while the others are under immense pressure from ongoing depleting ecological practices (Matthews et al., 2016). The resilience and sustainability of a system to natural disasters could be assessed through the assessment of the crossover of the nine planetary boundaries (Haffar, 2018). Hence, when assessing the sustainability and resilience of a system to natural disasters the planetary boundaries need to be considered. The KTSC is highly affected by natural disasters which are predicted to increase in the coming years (Reliefweb, 2021). At the same time, the current conceptualizations on the two phenomenon’s—resilience and sustainability—are inconclusive as they tend to consider, the focal firm or the industry, ignoring the global context (Whiteman et al., 2012). However, the planetary boundaries constraints on human development require a broader approach to be followed by organisations that integrates the macro ecological factors and their levels on a particular geographical area. Therefore, this research utilises the PBT to assess the sustainability—resilience relationship under the context of natural disasters in a particular geographical area i.e. KTSC. The PBP approach is used to assimilate the global operating environment, and thus inform on our understanding on how sustainability practices may affect resilience and vice versa in times of disruptions emanating from global operating environment factors. Therefore, the research gap that this research aims fill is the exploration of the relationship between sustainably and resilience practices and whether these can co-exist in the ASC from the PBT perspective. Based on the above the objectives of this research are:

1. Identify the current levels of planetary boundaries as well as sustainability and resilience levels in the Kenyan tea supply chain from the producers’ perspective.
2. Explore the relationship between sustainability and resilience under the planetary boundaries’ theory in the Kenyan tea supply chain from the producers’ perspective.

## Methods

### Research design and sample

The exploratory nature of this research and absence of research regarding the sustainability—resilience relationship in ASCs using the PBT calls for a multi-case study approach. This approach is appropriate as it enables an in-depth investigation of the phenomenon under study (Voss et al., 2016). The aim of this research is to develop theory about the relationship between sustainability and resilience in ASCs using the PBT by elaborating on the existing literature regarding sustainability and resilience in supply chains and ASCs, that is, ‘theory elaboration’ (Ketokivi and Choi, 2014). To this purpose, nine case studies of producer organisations have been included. One way that validity is ensured in this study is through the robust design of the interview questions (Table 7 in Appendix).

The case study approach was used to provide a qualitative basis and a deep understanding of the phenomena under investigation by examining the meanings that participants assign to them in a particular context (Orlikowski & Baroudi, 1991). The study herein cannot be separated from its original contextual environment (i.e. KTSC) and therefore used a cross-case analysis technique to identify relationships and compare them through interviews of multiple stakeholders (Eisenhardt, 1989; Yin, 2014) of different Kenyan tea supply chains. Also, another reason for applying the cross-case study approach is due to the research design, which allows concepts to emerge from the data but also allows the researchers to provide rival explanations from multiple stakeholder views (Yin, 2014). Miles and Huberman (1994) and Yin (2009a, b) use case studies as a preferred strategy when how or why questions are being posed, when the investigator has little control over events, and when the focus is on a contemporary phenomenon within some real-life context.

The data from the case study interviews were analyzed through the various phases of thematic analysis (Boyatzis, 1998; Braun & Clarke, 2006) and themes were generated based on the literature review (PBT elements, resilience practices, and sustainability practices), leading to an initial coding scheme (Table 7 in Appendix). Further thematic analysis was carried out to indicate the sub-themes and divide them into sub-groupings. Sample size is a difficult aspect to define for qualitative research, as it relies on the type of qualitative design (Creswell, 2013). For case study research, Creswell (2018) suggests to gather information from four to five cases to have rich data for analysis. However, Yin (2009a, b) argues that traditional criteria for deciding the size of sample in case study research are not applicable. Hence, this research is following the suggestion from Charmaz (2014) about the value of continue sampling until reaching saturation (i.e. when the information converges). Using that approach, this research has collected data from nine producer organizations for analysis. Nine in-depth interviews were conducted in total. The number of cases exceeds the threshold from Creswell (2018) and reaching saturation allowed us to gather enough information to provide meaningful results.

The tea producers have been identified initially through personal contacts and these were then used to create a further pool of respondents. The initial choice of informants provided a preliminary understanding of their awareness regarding PBT aspects, and their practices regarding resilience and sustainability. We endeavored to conduct interviews with producers likely to represent a diverse range of views regarding sustainability and resilience hence we included producers from different geographical regions in Kenya i.e. Kigoro, Kigumo, Mundoro, Kifere, Kanyoni, Gachege, Kiangynu, and Gahtanji.

### Data collection and analysis

There were two stages of data collection. First, to be able to understand resilience and sustainability practices in the Kenyan tea industry, in-depth semi-structured face-to-face interviews were conducted. Semi-structured interviews can be referred to as an approach that involves the interviewer preparing some predetermined set of questions but giving freedom to the respondent to add insights on issues that the interviewer did not consider in advance (Saunders et al., 2016). An interview protocol was developed based on the objectives of this study and it was used to keep the questions consistent across the different interviewees. The duration of the interviews varied from 40 to 45 min and the respondents agreed to be audio recorded. Informed consent forms and confidentiality agreements were given to each interviewee prior to interview. During the interview process the respondents were encouraged to elaborate on their answers and on anything that they considered to be relevant to this topic to explore in-depth any other issues. To validate the findings the interviewer also used observation as a data collection method necessarily to support his understanding of the different practices and to confirm that practices that had been mentioned by participants were being implemented on the ground.

Secondly, interviewees were invited to a focus group discussion. This discussion enabled the validation and triangulation of the findings. This research avoided bias by the triangulation of sources as the research used historical data to provide the background of each case before and after the interviews, observations of the processes and actions of the participants relevant to the objectives of this study and also the interviews with the participants. Interviews were conducted on several participants in different time frames and in varying locations. Credibility was further increased by selecting participants from the KTSC who had substantial experience in the industry and represented a valid picture of the research matter (Creswell, 2003). By informing the participants that the research information was strictly confidential and would only be used for this research project the researcher ensured that the participants were free to engage in a free way that enabled the researcher gain better insights.

Thematic analysis evidence has shown that the general trend is to begin with coding which then allows for themes to be generated (Miles & Huberman, 1994). Thematic analysis with coding was used to identify themes reflecting the interview guide. Two researchers coded each interview through NVivo Qualitative Data Analysis Software. After the end of each interview it was immediately transcribed to identify the need for further interviews and the transcripts were sent to the interviewees for checking. The results were reported under the main categories i.e. planetary boundaries, sustainability practices, and resilience practices.

To ensure transferability (equivalent to external validity), we followed Lincoln and Guba (1985) and provided sufficient information about the context of Kenyan supply chain where the research was conducted so that “anyone else interested in transferability has a base of information appropriate to the judgement” (pp. 124–125). To ensure credibility (equivalent to internal validity), we followed Lincoln and Guba (1985) and used interview data in parallel with observation, peer debriefing, and audit trails. Finally, we followed Lincoln and Guba (1985) and therefore the following strategies to ensure ruling out rival interpretations of data: (i) prolonged engagement with the organisation, understanding the culture and building trust with the participants (ii) persistent observations (iii) triangulation.

The results were presented in the focus group discussion for validation purposes.

## Findings

This section presents the findings of the primary data collected in order to understand the producers awareness of the existence of the PBT elements, to understand the resilience and sustainability practices employed with the aim to explore the relationship between sustainability and resilience in context of the KTSC guided by the PBT approach.

### Participants profiles

To preserve the anonymity of the participants, Table 2 presents a summary of this study’s participants based on the region and their experience in years. The table also indicates the participant’s codes, (denoted between P1 and P9). The number of years’ experience in the KTSC was thought to be an important metric as individuals with more experience would be able to compare experiences especially concerning climate change which is an ongoing occurrence. Validation of the data was achieved by triangulating the data collection across different regions—which were chosen because tea in these areas was the main economic activity.

**Table 2:** Participants details

Participants code	Experience in years	Region
P1	16	Kigoro
P2	33	Kigumo
P3	17	Mundoro
P4	33.5	Oleng
P5	20	Kaifere
P6	20	Kanyoni
P7	40	Gachege
P8	8	Kiangunu
P9	34	Gathanji

### Findings on planetary boundaries

The interviewed producers were asked about their awareness regarding the different planetary boundaries as presented in Table 7 in the Appendix. Most of the interviewees were aware about seven out of the nine planetary boundaries; they were not aware about novel entities and in stratospheric ozone depletion. Table 3 below summarizes the findings of the Planetary boundaries’ awareness per participant.

**Table 3:** Summary of findings about the planetary boundaries per participant

Participants code	Planetary boundaries awareness						
	Climatic change	Atmospheric aerosol loading	Land system change	Biochemical flows	Biosphere integrity	Fresh water availability	Ocean acid
P1	×			×			
P2	×			×			
P3	×		×		×		
P4	×						
P5	×	×	×				
P6	×		×		×		
P7	×		×				
P8	×		×	×	×	×	×
P9	×		×	×	×	×	

All interviewed producers were found to be aware of climatic change as they experience it highly. The impact of climatic change is mainly visible to them through the extreme weather conditions such as extreme rain, drought, and hail. Some of the interviewed producers stated that:

‟…for the last 3 years we have experienced extreme heat and drought and due to these it is really affecting tea production negatively.” (P1)

‟…a lot of rain makes the roots of the tea leaves freeze especially when the weather is misty. Also recently there were hail stones and because previously the weather was very sunny, the hail stones really affected the tea plantations.” (P3)

‟…the weather lately has been unpredictable not like in the earlier years, the atmosphere has really changed when we are expecting the rains it gets extreme hot and when we are expecting the sunny season it starts raining.” (P4)

Especially the hail and extremely cold weather seem to impact them significantly as it destroyed completely their produce. Extremely hot weather may impact negatively their production. Some of the interviewed producers mentioned that:

‟…When it is extremely hot we experience low production leading us to produce less kilograms, which can drop even to approximately 40%.” (P5)

In terms of the atmospheric aerosol loading, stratospheric ozone depletion, and novel entities only one of the interviewed producers was found to be aware of air pollution. Producer P5 mentioned that he is taking actions to reduce his emissions by measuring the CO_2_ emissions of his business. All the interviewed producers were found to use fertilizers which are ozone depleting substances and could possibly be considered as novel entities. However, none of them was aware of their negative implications on the environment. This is probably because they are given the fertilizers by their buyers as and they apply them directly to their produce without understanding what they are using. Since producers were not aware about the stratospheric ozone depletion and the novel entities planetary boundaries there are not included in Table 3.

Six out of the nine interviewed producers were aware of the land system change as it affects them as they frequently have landslides. One of the interviewed producers stated:

‟…this year we have had a lot of rains from March, April and May, because of the topography of this area being very hilly, we experienced a lot of landslides and some of our farmers lost their tea arising from the landslides. They wake up on morning and find that the farm is not there it has gone to the neighbors together with the tea. That is one area that has really been a problem.” (P7)

In response to the land system change they are adopting different measures to ensure high yields on their current land holdings in order to increase land productivity and improve environmental conditions such as training, dividing of their land holdings, and planting trees. Some of the interviewed producers mentioned:

‟…despite the fact that agricultural land has been decreasing what we have experienced is just farmers’ sub dividing their lands.” (P6)

‟…we have embarked so much on rehabilitating our rivers, riverbeds and planting of trees and that kind of programs. In fact, on Monday 16^th^, we are having a tree planting day.” (P8)

Only four out of the nine interviewed producers were aware of the biochemical flows planetary boundary which is related to nitrogen and phosphorus losses. These producers were found to be aware of the impact of their farming actions on the planet as rivers are impacted as well as the quality of their production. One the producers stated:

‟…there is a lot of heavy soil erosion such that rivers have turned almost yellow because of the soil. Thirdly, due to the excessive rains the crop has been affected negatively because when there is too much water in the soil this reverses the gains that we have had. Additionally, the fertilizers that we do buy we have not seen the results because the soils are washed out too much by the rains.” (P8)

In terms of changes in biosphere integrity which is about awareness of biodiversity implications on their farming business only a few producers were aware of it. This is evident to them as their yields are decreasing, and new infections and other plant types appear and damage their production. This has further implications as tea producers may choose to invest in other types of crops that are more profitable and future tea production may be impacted. Some of the interviewed producers stated:

‟…As a result of soil erosion, tea plants are no longer producing especially during the dry season. With dry season we have lost crops, sometimes even bushes have dried up, we have also lost our young seedlings.” (P9)

‟…we opt uprooting the tea plantations when they dry up due infections and plant other types of cash crops.” (P3)

Only two out of the nine interviewed producers were aware of the freshwater availability due to negative implications on their produce as current water resources seem not to be sufficient for them. A few of the interviewees mentioned:

‟…during extreme hot weathers, we face a lot of challenges since we can’t water the plantations because the water is rationed, and the water might not be sufficient to everyone. We therefore depend on purely rain fed agriculture. So, if we don’t receive adequate rainfall, production goes down.” (P2)

‟…on water management, us farmers in this area we don’t water our tea farms since there is no sufficient water supply. If tea farming had better returns, we would risk to get water from somewhere else at a cost, but now we don’t get much from tea farming therefore we can’t afford to incur others costs lest we work at a loss.” (P4)

There were a few of them that they are implementing water management systems and they hold certifications such as the Certification of the Rain Forest Alliance in their efforts to save water and use it more efficiently. Only one of the interviewed producers was found to be aware of the ocean acidification issue. This is probably because it does not seem to affect them directly as their landholdings are on highlands. One of the interviewed producers stated:

‟…on ocean acidification, that’s a non-issue to us because our farms are in the highlands and also when we apply fertilizer we do so when there is minimal rain and by the time the rains come, the plant has already utilized the fertilizer.” (P8)

### Supply chain sustainability findings

This section presents the findings of the three sustainability elements in terms of the different practices that the interviewed producers employ. Most of the interviewees were found to be engaged in at least two sustainability practices (Table 4).

**Table 4:** Summary of findings about sustainability practices per participant

Participants code	Sustainability practices		
	Environmental	Social	Economic
P1	×		×
P2	×	×	
P3	×		
P4	×	×	
P5	×		×
P6	×	×	×
P7	×	×	×
P8	×	×	×
P9	×	×	×

Eight out of the nine interviewees implement environmental sustainability practices. These practices are related to the use of water meters, energy saving equipment and other alternative energy methods, as well as waste management. A few of them have acquired the Rain Forest Alliance certification for water preservation purposes. One of the producers claimed:

‟…the Certification of the Rain Forest Alliance is one of the key criteria in water management. That is why we are having a lot of programs together with other stakeholders like Water Resources Management Authority. We are going back to our rivers; we are having policies and active participations in protecting our rivers.” (P8)

Producers are receiving training about waste and water management and they were found to have recycling schemes for plastic recycling. Any food waste is used as manure in their produce. Some of the interviewed producers stated:

‟…people inspecting on waste management make sure the pits have been dug in certain specifications and that they are big enough to hold quite a number of these polythene. (P2)

‟…yes, we have been taught on garbages, things like plastic paper bags are supposed to be collected and recycled and not burnt or buried.” (P3)

They are also investing in alternative sources of energy to firewood as they found out that it is not sustainable due to the tree cutting that it requires and the greenhouse gas emissions that are generated. The use of firewood generates further CO_2_ emissions when the trees are burnt in the boiler. None of the interviewed producers were found to currently have any practices about reducing greenhouse gas emissions.

Although there were no visible alternative sources on display at the time, two producers mentioned that they were considering the use of solar systems to replace their current energy sources. But these plans were at an initial stage as electricity and firewood were the main source of energy. Although the purchase of land to plant more trees for firewood while they were considering an alternative source of fuel seemed counterproductive and the solution to that is to plant more trees for environmental conservation. One of the interviewed producers mentioned:

‟…we are also researching to know what else we can do or what else we can use as a source of energy, since firewood is our main source of energy that’s what we use here for generating steam. I know it has an effect on the climate since the more we cut trees the more we are affecting the rainfall and also emitting a lot of greenhouse gases. We are encouraged to plant trees, and we are buying farms to plant our own trees for firewood that will act as a source of energy.” (P6)

Six out of the nine producers were found to be engaged in social sustainability practices. These practices are focussed on child labour prohibition and on promoting non-gender and non-racial discrimination. An interesting observation was that only women seemed to be working as tea pickers in the farms as men do not like to work on that.

‟…there is no discrimination when employing farm workers, though most of our workers are women compared to men. Regarding employing children to work on the farms, we have been educated by KTDA not to employ any child since that is child labour and it’s against the law.” (P4)

Producers also receive training about waste management, and environmental protection from their buyers. One of them state:

‟… we receive training from our buyers on how to handle the waste in farms, water and all the waste that is generated in the farm. We separate the wastes then they collect them from their farms.” (P6)

Six out of the nine producers claimed to be economically sustainable at the moment, but they are not sure if this will continue in the future. Although the Kenyan tea industry pays the most to producers among competing countries, all producers felt that they were not being paid enough to sustain their business and they are considering of investing in other types of produce that are more profitable. One of the producers stated:

‟…as a tea farmer, since we are not getting sufficient money from tea farming, some are opting planting avocado trees and macadamia since the farms are small which have a better income and also there is manpower involved like you don’t have to employ workers unlike tea farming. When these avocado and macadamia are ready for harvest, the buyers come to harvest for themselves unlike tea whereby we farmers pluck ourselves.” (P4)

Pay on the farms was based on a piecemeal basis that paid the worker on output and depending on how much tea one was able to pick then there was a standard price per kilo. The standard rate per kilo of raw tea leaves seemed to have been a factor of market economics as they all did not know how it was agreed upon and was more instinctive rather than planned. One producer mentioned:

‟…around this area we pay our workers 10 shilling per kilogram. Though at times we are straining because, at the factory we are paid 15 shillings per kg then I pay my worker 10 shilling per kilogram, the remainder is 5 shillings then you less the fertilizer, less manpower to take care of the farm. Most of us are forced to rely on bonuses only.” (P5)

Therefore, in terms of the sustainability practices the majority of the interviewed producers were found to implement environmental sustainability practices, while almost half of them have in place social economic sustainability practices (6 social and 6 economic). Four of the interviewees were found to have adopted practices related to all the three sustainability elements (i.e. environmental, social, and economic).

### Supply chain resilience findings

This section presents the findings based on the three aspects of ASC resilience as identified in the literature review i.e. readiness, response and adaptability. Table 5 presents a summary of the findings about resilience practices per participant.

**Table 5:** Summary of findings about ASC resilience aspects per participant Participants Code

	Resilience practices		
	Readiness	Response	Adaptability
P1		×	
P2	×		
P3			×
P4			×
P5			×
P6	×		×
P7			×
P8	×	×	×
P9	×	×	×

Regarding the readiness aspect of resilience, four out of the nine interviewees were found to implement readiness practices in case of a disruption due planetary boundaries implications. Most of them they keep extra stock, postponement activities, and conduct risk assessments. Some of the interviewed producers mentioned:

‟…we keep extra produce in our warehouses in case there is a disruption. To achieve that we collaborate with buyers in order to store even more products.” (P8)

‟…we do risk assessment and then we have a list of all the risks that are likely to happen then put the mitigation factors which we put in place just in case that risk happens.” (P6)

In terms of response, only three out of the nine respondents found to have response practices in case of a disruption. These practices are about information sharing, development of collaborative capabilities with key buyers, and training about responding to unexpected situations.

‟…yes, we have never shut down, we are able to deal with the challenges as they come. We share information with our collaborators to understand how to deal better with it.” (P8)

‟…through the collaborative activities with our buyers we are taught about how to best response in different kinds of unexpected situations.” (P2)

Most of the interviewees i.e. seven out of the nine stated that they implement adaptability practices. This indicates that most of them wait until the disruptions happens and then they try to adapt to the changing circumstances such as adapting to changing weather conditions and plant more tea trees instead of investing in building readiness and response capabilities. This could be considered as a more passive approach to resilience.

‟…we don’t do much, when the tea leaves are destroyed by for example the hail stones, we just prune them and wait for the tea leaves to shoot again. There is nothing we can do since it’s a natural calamity.” (P4)

‟…I think I am well prepared for climate change, though not 100% but we have mitigation issues that are there in place, like what I said we encourage all the producers to plant a lot of trees. By this we reduce the rate at which the climate is going to affect us.” (P6)

Hence, it was found that the majority of the interviewed producers invest in the development of adaptability capacities, then to readiness capabilities and only a few of them to response capabilities.

### Supply chain sustainability and resilience findings

For the purposes of this analysis the sustainability and resilience practices of the interviewed producers were ranked as: (a) low implementation (L: only one sustainability or resilience practice in place), (b) average implementation (A: two sustainability or resilience practices in place), and (c) high implementation (H: all three sustainability or resilience practices are in place). A similar categorization was followed to rank the PBT awareness findings and this is: (a) low awareness of PBT (L: aware of only one to two planetary boundaries), (b) average awareness of PBT (A: aware of three to four planetary boundaries), and (c) (b) high awareness of PBT (H: aware of more than four planetary boundaries). Table 6 presents a summary of this study’s findings and the respective rankings. The rankings for the awareness of Planetary boundaries are presented as PR, for the resilience practices as RR, and for the sustainability practices as SR.

**Table 6:** Summary of this study’s findings about the relationship between sustainability and resilience under the PBT

PC	Planetary boundaries awareness								Resilience practices				Sustainability practices			
	CC	AL	LC	BF	BI	FW	OA	PR	RD	RS	AD	RR	EN	SO	EC	SR
P1	×			×				L		×		L	×		×	A
P2	×			×				L	×			L	×	×		A
P3	×		×		×			A			×	L	×			L
P4	×							L			×	L	×	×		A
P5	×	×	×					A			×	L	×		×	A
P6	×		×		×			A	×		×	A	×	×	×	H
P7	×		×					L			×	L	×	×	×	H
P8	×		×	×	×	×	×	H	×	×	×	H	×	×	×	H
P9	×		×	×	×	×		H	×	×	×	H	×	×	×	H

Overall, the interviewed producers were found to implement more sustainability practices than resilience practices. Therefore, being sustainable does not mean that they will be resilient at the same time. Four of them were ranked as having high implementation of sustainability practices, four average, and one low. Six out of the nine interviewees ranked low in terms of their resilience practices implementation as they were found to implement only one of the three resilience aspects, one of them average, and two of them as high.

Regarding the relationship between sustainability and resilience, most of the producers who ranked high or average on sustainability practices implementation, they have average or low rankings for resilience practices implementation. In particular these are: P1, P2, P4, P5, P6, and P7. In these cases, it seems that the interviewed producers implement sustainability practices, but this affects their implementation of resilience practices. However, sustainability is increasing faster than resilience. This may suggest the existence on a non-linear relationship among sustainability and resilience. Regarding the PBT it was found that when there is low or average awareness of the PBT constraints the resilience practices implementation is low or average as well (P1, P2, P4, P6, P7). However, sustainability practices implementation does not seem to be affected by the producers’ awareness of the PBT factors. This suggests that PBT factors awareness is related to increased resilience but does not seem to affect sustainability practices implementation. Therefore, the PBT factors awareness was found to impact resilience practices implementation.

## Discussion

The findings identified that ecological conditions were posing a real challenge to producers. Tea growing areas had started to experience climatic conditions that had not been experienced before, for instance hailstones had started to appear just five years ago as indicated by the findings. This is evidence of loss of stability by the ecosystem which is in line with Rockström et al. (2009) theory on the safe operating spaces. The analysis pointed out that the main issue affecting tea production was climatic change as producers are highly impacted by that. Most of the Kenyan tea producers were found to be aware of the following planetary boundaries: climatic change, land system change, biochemical flows, and biosphere integrity. Only one producer was found to be aware of the atmospheric aerosol loading boundary although the extensive use of pesticides on their produce affects this boundary. Awareness about the ocean acidification boundary was mentioned by only one producer. Interestingly, the novel entities and the stratospheric ozone depletion boundaries were not familiar to producers. This may be because they collect the chemicals and the pesticides that they use from their buyers, hence they are unaware of the products that are using and their negative implications on the environment. Therefore, regarding the awareness of the PBT this research adds to the existing literature of Rockström et al. (2009) and Whiteman et al. (2012), that these boundaries exist in the ASC and although certain boundaries might have been already crossed the KTSC producers were aware of only a few of them. Therefore, increasing awareness activities at all the levels of the ASC is important.

Regarding the sustainability practices implementation this research this study found that some producers participate in programs to manage waste, water and utilise energy in an efficient way. However, greenhouse gas emission was not being addressed and there was little investment on alternative clean energy sources. At the producer level, waste, water, and energy were all being managed but greenhouse gas emissions and alternative energy were not being practiced. The study exhibited environmental sustainability conformity which is in line with Elkington (1998) and Despoudi (2020), but the lack of greenhouse gas emission management and limited investment on alternative energy sources went against the literatures recommendations. Social sustainability requires that organisations engage in fair labour practices and safeguard human rights (Elkington, 1998). This study found evidence of social sustainability practices in the KTSC at the producers’ level. Producers portrayed evidence in the engagement of social issues such as safeguarding against discrimination and fair compensation. Therefore, although there were issues that were in line with Elkington (1998) recommendations of social sustainability practices, some other issues especially at the producer level were less sustainable in relation to the literature.

Economic sustainability advocates for profit, cost management, share price growth and revenue growth (Elkington, 1998). Producers felt that they were not making enough money from tea as compared to alternative types of crops. At the same time, they indicated that their costs had substantially gone up, and they were not making enough money from tea to keep up. Therefore, although there was indication about current economic sustainability in terms of the future is seems quite unsustainable. Overall, almost half of the interviewed producers were found to have high or average implementation of sustainability practices with all of them having environmental practices in place.

This study adopted the stages of disruptions to identify the right capabilities at each stage as suggested by Stone and Rahimifard (2018) that had been developed by Ponomarov and Holcomb (2009) and Hohenstein et al. (2015). In terms of the readiness capability, this study found that some producers had built redundancy and flexibility capabilities in their operations which is in line with the research of Christopher and Peck (2004). Flexibility was also evident in the KTSC as producers developed strategic partnerships with their buyers which is in line with the examined literature (Christopher & Peck, 2004; Ponomarov & Holcomb, 2009). The findings of this study suggested that collaboration is well practiced by producers and there was evidence of existing links of communication and information sharing between producers and their buyers and how this had been used in times of disruptions. This adds to the existing research of Dubey et al. (2020) and Faisal et al. (2006) about the response capabilities of visibility through information sharing and collaboration by replicating this finding in the KTSC context. Most of the producers were found to implement adaptability practices through flexible sourcing and this confirms the findings of Stone et al. (2015a; b). Overall, producers failed to show strong implementation resilience practices to quantify them as being very resilient as per the resilience literature as they exhibited mainly adaptability practices which could be considered as a more passive approach to resilience.

### Research implications

The findings of this study indicated that sustainability and resilience are interrelated concepts. This in line with the fourth perspective about the sustainability resilience relationship as suggested by the authors of this study in Table 1 and the studies of Higgins et al. (2010) and Mohammed et al. (2021). However, the findings of this study suggest that the sustainability—resilience relationship is non-linear. As although the concepts were found to be related, they do not seem to increase at the same pace. This adds to the existing literature of Marchese et al. (2018) and Colwill et al. (2016) that there is a fourth perspective of the latter relationship but it is not linear in the KTSC context. They are related concepts but the way the one increases does not impact the other in the same way. However, the type of non-linear relationship should be further examined by future research.

Regarding the PBT it was found that when there is low or average awareness of the PBT constraints the resilience practices implementation is low or average as well (P1, P2, P4, P6, P7). However, sustainability practices implementation does not seem to be affected by the producers’ awareness of the PBT factors. This suggests that PBT factors awareness is related to increased resilience but does not seem to affect in the same way sustainability practices implementation. Therefore, the PBT does not affect in the same way sustainability and resilience. The findings of this study suggested that there is a relationship between resilience practices and PBT factors awareness, however there is no relationship between sustainability practices implementation and PBT awareness. This adds to the research of Haffar (2018) who claimed that there is a relationship of PBT with both sustainability and resilience, by suggesting that in the KTSC context this is not the case. Therefore, to understand the latter relationship specific contextual factors need to be studied. Hence, the propositions resulting from this study are:

P1: The relationship between sustainability, and resilience is non-linear.

P2: When the awareness about the PBT constraints is high, resilience will be higher and when awareness is low, and resilience will be low.

### Managerial implications

This study presents some useful insights for managers and other ASC entities. Firstly, having evaluated the current status of the tea industry in Kenya, this research could encourage ASC managers and other ASC entities to critically evaluate their sustainability and resilience approaches in the face of vulnerabilities – PBP in this case. Secondly, this research illustrated how producers within the same supply chain may employ diverse resilience and sustainability practices and indicated the practices that are mostly used. Hence, ASC managers and producers could learn from each other and synergies could be enabled to build sustainable and resilience ASCs in the face of disruptions.

### Generalisability of results

The purpose of case study research is to gain deeper understanding about processes, the context and the perception from stakeholders (Benbasat et al., 1987) and it cannot be reduced to a series of generalisations (Saunders et al., 2009). Indeed, case study analysis is not intending to generalise from limited samples to the entire population (Yin, 2009a, b), but to provide thorough understanding complex environments such as the relationship between sustainability and resilience using PTB. Hence, case studies do not allow statistical generalisability (Yin, 2009a, b). However, they allow us to create theoretical premises to make assertions about similar situations to the one studied (Wikfeldt, 1993). In fact, the concept of analytical generalisation arises (Gomm et al., 2009; Yin, 2009a, b), which involves considering several case studies with similar characteristics and results as basis to develop theory. This concept has also been described as theoretical generalisation (Tsang, 2014). As a result, the theoretical insights from this research can be used to inform tea supply chains in similar countries and to be used as basis for the development of similar studies in different countries to develop theoretical generalisations.

## Conclusions, limitations and future research

The aim this study was to evaluate whether a supply chain could simultaneously tolerate economic growth, balance social and environmental impacts and still be resilient to disruptions by examining the relationship of resilience and sustainability in the KTSC under the PBT. Therefore, this study sought to understand the current practices in the industry by interviewing nine producers who were dependent on tea for their livelihoods. Through thematic analysis of the primary data from the interviews, insights were generated about the current PBT awareness levels, current resilience and sustainability practices, and the relationship between sustainability under the PBT in the KTSC. The findings suggest that KTSC producers are aware of most of the planetary boundaries and they are engaged in sustainability and resilience practices. Although sustainability and resilience were found to be interrelated concepts, they are not increasing in the same pace. Hence a non-linear relationship was detected. Further research is needed to ascertain the type of non-linear relationship between sustainability and resilience.

Also, the PBT awareness factors were found to be linked to resilience practices and not to sustainability ones. This research used interviews from a specific population of interest i.e. KTSC producers. Future studies should use other forms of data collection methods such as questionnaires to get more generalisable results and collect data from other ASC entities i.e. processors, retailers, and other types of ASCs and explore further the awareness of the PBT factors. This research adopted a case study approach which was focused on the Kenyan tea producers only and hence the generalisability of the results is limited to this specific context. Future research should replicate the findings of this study to other ASCs in other countries. Due to the study’s objective to explore the phenomenon’s key practices and explore the potential relationship between sustainability and resilience, this study has not laid down a detailed framework on how organizations could be able to achieve a balance of resilience and sustainability. Thus, further research is needed to develop a comprehensive framework of how sustainability and resilience could be balanced and used by organisations.

## Appendix

See Appendix Table

**Table 7:** Summary of concepts, abbreviations, definitions, and sources.

Concept	Abbreviation	Definition	Source
Climatic change	CC	the extent to which the organization is aware of the implications of climatic change e.g. emissions, energy imbalance	Based on Rockström et al. (2009)
Atmospheric aerosol loading	AL	the extent to which the organization is aware of the atmospheric aerosol boundary/issue (i.e. air pollution)	Based on Rockström et al. (2009)
Stratospheric ozone depletion	SD	the extent to which the organization is aware of their impact on Stratospheric ozone Depletion	Based on Rockström et al. (2009)
Land system change	LC	the extent to which the organization is aware of the global land cover converted to cropland	Based on Rockström et al. (2009)
Biochemical flows	BF	the extent to which the organization is aware of their impact on the biochemical flows’ boundary related to its control and losses of nitrogen, phosphorus, and other biochemical flows	Based on Rockström et al. (2009)
Change in biosphere integrity	BI	the extent to which the organisation is aware of the implications of biodiversity on their business as i.e. functional well as their impact on biodiversity loss	Based on Rockström et al. (2009)
Novel entities	NV	the extent to which the organisation uses novel entities e.g. chemicals, new types of engineering materials/organisms	Based on Rockström et al. (2009)
Fresh water availability	FW	the extent to which the organisation uses freshwater in a sustainable way	Based on Rockström et al. (2009)
Ocean acidification	OA	the extent to which the organisation is aware of the ocean acidification boundary/issue	Based on Rockström et al. (2009)
Readiness	RD	the extent to which an organization anticipates or prepares for a potential disruption	Based on Stone and Rahimifard (2018)
Response	RS	the extent to which an organization has pre-planned elements that mitigate the impact of a disruption and help it to remain functional	Based on Stone and Rahimifard (2018)
Adaptability	AD	the extent to which an organization has the capability to adjust is operations in response to a disruption	Based on Hohenstein et al. (2015)
Resilience ranking	RR	overall ranking based on RD, RS and AD	N/A
Environmental sustainability	EN	the extent to which an organization engages in environmentally friendly practices	Elkington (1998)
Social sustainability	SO	the extent to which an organization engages in social practices	Elkington (1998)
Economic sustainability	EC	the extent to which an organization is economically performing well	Elkington (1998)
Sustainability ranking	SR	overall ranking based on EN, SO and EC	N/A

7.
